# High-quality genome assembly of the soybean fungal pathogen *Cercospora kikuchii*

**DOI:** 10.1093/g3journal/jkab277

**Published:** 2021-08-05

**Authors:** Takeshi Kashiwa, Tomohiro Suzuki

**Affiliations:** 1 Biological Resources and Post-harvest Division, Japan International Research Center for Agricultural Sciences (JIRCAS), Tsukuba, Ibaraki 305-8686, Japan; 2 Center for Bioscience Research and Education, Utsunomiya University, Utsunomiya, Tochigi 321-8505, Japan

**Keywords:** *Cercospora kikuchii*, soybean, Cercospora leaf blight, purple seed stain

## Abstract

Plant diseases caused by the *Cercospora* genus of ascomycete fungi are a major concern for commercial agricultural practices. Several *Cercospora* species can affect soybeans, such as *Cercospora kikuchii* which causes soybean leaf blight. Speciation in *Cercospora* on soybean has not been adequately studied. Some cryptic groups of *Cercospora* also cause diseases on soybean. Moreover, it has been known *C*. *kikuchii* population genetic structure is different between countries. Consequently, further genomic information could help to elucidate the covert differentiation of *Cercospora* diseases in soybean. Here, we report for the first time, a chromosome-level genome assembly for *C*. *kikuchii*. The genome assembly of 9 contigs was 34.44 Mb and the N50 was 4.19 Mb. Based on *ab initio* gene prediction, several candidates for pathogenicity-related genes, including 242 genes for putative effectors, 55 secondary metabolite gene clusters, and 399 carbohydrate-active enzyme genes were identified. The genome sequence and the features described in this study provide a solid foundation for comparative and evolutionary genomic analysis for *Cercospora* species that cause soybean diseases worldwide.

## Introduction

*Cercospora* is one of the major groups of plant pathogenic fungi and *Cercospora* spp. can cause necrotic leaf spots on many different species of plants (Groenewald *et al.* 2013). *Cercospora kikuchii* (Tak. Matsumoto & Tomoy.) M. W. Gardner is a soybean pathogen, identified in eastern Asia in the early 20th century (Suzuki 1921; Matsumoto and Tomoyasu 1925; Walters 1980). It causes two types of pathogenic symptoms in soybean: purple seed stain (PSS) on seed pods and seeds, and Cercospora leaf blight (CLB) on leaves and petioles. Both symptoms present a typical dark-purple-colored lesion. This is caused by cercosporin, a pigment produced by the pathogen. The pigment induces cell death of the host plant in conditions with light (Kuyama and Tamura 1957; Yamazaki *et al.* 1975; Daub and Hangarter 1983).

Symptoms caused by *C*. *kikuchii* are frequently observed in soybean fields. Currently, CLB is one of the biggest problems for soybean production in many areas of South America, such as Argentina (Wrather *et al.* 2010). It has been suggested that the *C. kikuchii* populations in South America and Japan are different, based on phylogenetic analysis (Imazaki *et al.* 2006). It has also been reported that other *Cercospora* species such as *C*. cf. *flagellaris*, *C*. cf. *sigesbeckiae* (Albu *et al.* 2016), and *C*. cf. *nicotianae* (Sautua *et al.* 2020) can also cause soybean leaf blight. Moreover, some cryptic species of *Cercospora* infect soybean (Soares *et al.* 2015). Phylogenetic studies of *Cercospora* species, including CLB pathogens, have been reported (Groenewald *et al.* 2013; Soares *et al.* 2015). Such investigations will significantly benefit from additional high-quality genomic resources.

The genomes of the *Cercospora* spp. such as *C*. *zeae*-*maydis* (causal agent of gray leaf spot on corn, Haridas *et al.* 2020) and *C*. *beticola* (causal agent of leaf spot on sugar beet and other plant species, Vaghefi *et al.* 2017) have been analyzed and deposited in public databases. Additionally, genome sequences of the species infecting soybean have been published, such as *Cercospora sojina* (frogeye leaf spot, Luo *et al.* 2018; Gu *et al.* 2020) and *Cercospora* cf. *sigesbeckiae* (leaf blight, Albu *et al.* 2017). For *C*. *kikuchii*, two genomes have recently been deposited. These genomes were obtained from isolates originating from the United States (isolate A3, NCBI GenBank accession: GCA_005356855.1, contig N50: 364,948 bp) and Argentina (isolate ARG_18_001, NCBI GenBank accession: GCA_009193115.1, Sautua *et al.* 2019, contig N50: 675,846 bp), but have low contiguity.

The aim of this study was to obtain a reference genome sequence for *C*. *kikuchii* for further comparative genomic studies of the *Cercospora* species. The Japanese *C*. *kikuchii* isolate MAFF 305040 was sequenced. The isolate was subjected to species-wide phylogenetic study of the genus *Cercospora* in the previous study (Groenewald *et al.* 2013). Sufficient depth of sequencing coverage by long-read sequences generated a chromosome-level assembly. This high-quality genome sequence data provide fundamental genomic information and deepen our understanding of the pathogen.

## Materials and methods

### Fungal strain and culture conditions

*Cercospora kikuchii* MAFF 305040 was provided by the National Agriculture and Food Research Organization (NARO) Genebank (Tsukuba, Ibaraki, Japan). The isolate was maintained and cultured by following the method (Kashiwa *et al.* 2021).

### DNA extraction from the fungus

Mycelia were collected from 3- to 4-day-old liquid cultures and dehydrated on paper towels. Collected mycelia (approximately 1 g) was ground in liquid nitrogen and extracted with 10 mL of CTAB lysis buffer (100 mM Tris-HCl, 2% CTAB, 1.4 M NaCl, 10 mM EDTA) at 65°C for 1 h. DNA was purified using a double extraction by mixing for 15 min with an equal volume of chloroform: iso-amylalcohol mixture (24:1) and then centrifuged at 8,370 × *g* for 10 min. The DNA was precipitated with ethanol and then centrifuged at 20,400 × *g* for 5 min at 4°C. The pellet was washed with 70% ethanol and dried. The DNA was resuspended in 10 mM Tris-HCl solution and stored at −30°C prior to genome sequencing.

### Genome sequencing and data manipulation

The genome DNA library was prepared and subjected to sequencing by PacBio RS II (Pacific Biosciences, Menlo Park, CA, USA). RNA removal from the DNA solution, DNA library preparation, and sequencing was performed by Macrogen Japan (Tokyo, Japan). Statistics for the sequence data was calculated by SeqKit 0.12.1 (Shen *et al*. 2016). The obtained sequence data were assembled by Canu 1.9 (Koren *et al.* 2017). Default values for Canu were kept for the assembly step. The estimated genome size was set to 34 Mb. Assembled sequences were polished by Arrow 2.3.3 (Pacific Biosciences). The polished assembly was checked by Tapestry 1.0.0 (Davey *et al.* 2021) with default values. The telomeric repeat TTAGGG (Cohn *et al.* 2005) was for the detection of telomeric repeats. The software Tapestry is also used for the evaluation of read depth and GC content of each contigs.

The Dfam TE Tools 1.4 for Docker was used for soft masking the genome based on *de novo* repeat finding by RepeatModeler (Flynn *et al.* 2020). The container incorporating the programs obtained from http://www.repeatmasker.org/ (RepeatModeler, RepeatMasker, coseg, RMBlast, RepeatScout, and RECON) and other tools such as HMMER (http://hmmer.org/), CD-HIT (Fu *et al.* 2012; Li and Godzik 2006), GenomeTools (Gremme *et al.* 2013), LTR_retriever (Ou and Jiang 2018), MAFFT (Katoh and Standley 2013), NINJA (Wheeler 2009), and Tandem Repeats Finder (Benson 1999). Gene prediction was performed using BRAKER2 (braker.pl version 2.1.6; Hoff *et al.* 2016; Hoff *et al.* 2019; Brůna *et al.* 2021) incorporating Augustus 3.4.0 (Stanke *et al.* 2006; Stanke *et al.* 2008). Genes were predicted with proteins of any evolutionary distance (--epmode). The fungal protein database was obtained from OrthoDB (https://v100.orthodb.org/download/odb10_fungi_fasta.tar.gz, accessed on August 24, 2020) and subjected to gene prediction using the ProtHint pipeline (Lomsadze *et al.* 2005; Iwata and Gotoh 2012; Gotoh *et al.* 2014; Buchfink *et al.* 2015; Brůna *et al.* 2020) in BRAKER2. Genes predicted by Augustus with hints were used for downstream analysis. Completeness of the genome assembly was assessed by BUSCO v4.1.2 (Seppey *et al.* 2019) for the predicted proteins, using the dataset dothideomycetes_odb10 (2020-08-05) obtained from the database.

Secreted proteins were predicted using the deep learning-based program DeepSig (docker image: bolognabiocomp/deepsig:latest, accessed on June 9, 2021; Savojardo *et al.* 2018), then candidate effector proteins were predicted using EffectorP 2.0 (Sperschneider *et al.* 2018) from the proteins predicted to have signal sequence at the N-terminus. Prediction of the carbohydrate-active enzymes (CAZymes) was performed using the dbCAN meta server (Yin *et al.* 2012; Zhang *et al.* 2018; http://bcb.unl.edu/dbCAN2/, accessed on June 9, 2021). The result of HMMER was used when other tools (DIAMOND and/or Hotpep) also predicted the protein as CAZymes. Secondary metabolite (SM) gene clusters were predicted using antiSMASH 5.1.1 (Blin *et al.* 2019). Genome information was visualized using Circos v0.69-8 (Krzywinski *et al.* 2009).

### Comparative analysis with the *C*. *sojina* genome

The genome assembly of the *C*. *sojina* Race 15 (Gu *et al.* 2020, NCBI GenBank accession: GCA_004299825.1, accessed on September 3, 2020) was obtained from the database. The genome assembly was subjected to gene prediction and annotation following the aforementioned scheme that was used for the MAFF 305040 genome.

## Results and discussion

### Assembly and completeness of the genome sequence

Genome sequencing generated more than 3.5 Gb from 3 SMRT cells of the PacBio RS II. Read length N50 was approximately 14 kb. The polished genome assembly for MAFF 305040 consisted of 15 contigs comprising approximately 34.55 Mb. GC content of the polished assembly was 53.0%. Based on the size of contigs, we obtained nine large contigs (2.36–5.86 Mb) and six small contigs (1.2–54.3 kb). To filter contigs, we used two criteria based on Tapestry results: contig GC content ranges 53 ± 10%; and median of read depth for contig is above 20. After filtering, six small contigs were excluded. Telomeric repeats were detected on both ends for eight contigs out of the nine selected contigs. This suggested that the eight contigs were close-to-complete chromosomes. The nine contigs comprising approximately 34.44 Mb were subjected to downstream analysis (Table 1). GC content of the nine contigs was 53.0% (Table 1).

**Table 1:** Summary of the genome assembly

Features	Value
Isolate name	MAFF 305040
Culture collection	NARO Genebank, Japan
Origin of isolate*^a^*	Kagoshima, Japan
Assembly size (bp)	34,440,063
Number of contigs	9
Maximum contig length (bp)	5,855,908
N50 of contigs	4,190,438
GC content (%)	53.0
BUSCO completeness (%)	99.4
Predicted genes	13,001
Genome accession*^b^*	BOLY00000000

a Data obtained from NARO Genebank (https://www.gene.affrc.go.jp, accessed on August 31, 2020).

b DDBJ/EMBL/GenBank accession number for the assembled contigs.

As a result of repeat masking, approximately 1.45 Mb (4.21% of total sequence) was masked. Among the masked regions, 119 LINEs, 426 LTR elements, and 92 DNA transposons were detected.

Recently, genome sequences of soybean pathogens were deposited in public databases, and some of their assemblies produced chromosome-level contigs. Compared to *C*. *sojina* (12 chromosomes comprising approximately 40.12 Mb, NCBI GenBank accession: GCA_004299825.1, Gu *et al.* 2020), the genome of *C*. *kikuchii* MAFF 305040 was found to be somewhat smaller, while that of *C*. cf. *sigesbeckiae* (approximately 34.94 Mb, NCBI GenBank accession: GCA_002217505.1, Albu *et al.* 2017) was of a similar size. However, consideration should be given to the fragmentation of the *C*. cf. *sigesbeckiae* genome (1,945 contigs) for size comparison.

A total of 13,001 genes were predicted by Augustus that was trained by the BRAKER2 pipeline. The pipeline used a fungal gene ortholog database, and Augustus was trained by the proteins from any evolutionary distance for gene prediction. Based on the predicted coding protein sequences, the completeness of the genome assembly was estimated to be 99.4% by BUSCO (Table 1). In the 3,786 groups, there were only 21 missing and 4 fragmented BUSCOs for MAFF 305040.

### Pathogenicity-related genomic features and comparisons to *C*. *sojina*, one of the soybean pathogen of genus *Cercospora*

To obtain information about the pathogenicity-related genes in the genome, several programs were used for functional annotation. Effectors are an important feature of plant pathogenic fungi, as they suppress host immunity. Effector proteins possess several characteristics, such as being small, secreted, and they have a high proportion of cysteine residues (Sperschneider *et al.* 2018). The DeepSig program estimated that a total of 1,393 proteins harbored an N-terminus signal peptide sequence for secretion. From the predicted secretome proteins, the EffectorP 2.0 program identified 245 proteins as effector candidates. The number of the genes for effector candidates was 242 (Table 2) after removing duplicated predictions for two transcripts from one gene.

**Table 2:** Candidates for pathogenicity-related genes and gene clusters

Features	Number
Effector candidates*^a^*	242
SM gene clusters*^b^*	55
Polyketide	15
Nonribosomal peptide	22
Terpene	6
Beta-lactone	1
Siderophore	2
Fungal-RiPP	1
Others	8
CAZymes*^c^*	399
GH	220
GT	86
CBM	10
AA	66
CE	19
PL	7

a Genes encoding effector candidates predicted by EffectorP.

b Numbers of SM gene clusters for each metabolite type predicted by antiSMASH. Fungal-RiPP, fungal ribosomally synthesized and post-translationally modified peptide.

c Genes encoding carbohydrate-activate enzymes (CAZymes) predicted by dbCAN. Some genes were annotated with more than one category. GH, glycoside hydrolase; GT, glycosyltransferase; CBM, carbohydrate-binding module; AA, auxiliary activity; CE, carbohydrate esterase; and, PL, polysaccharide lyase

**Figure 1:**
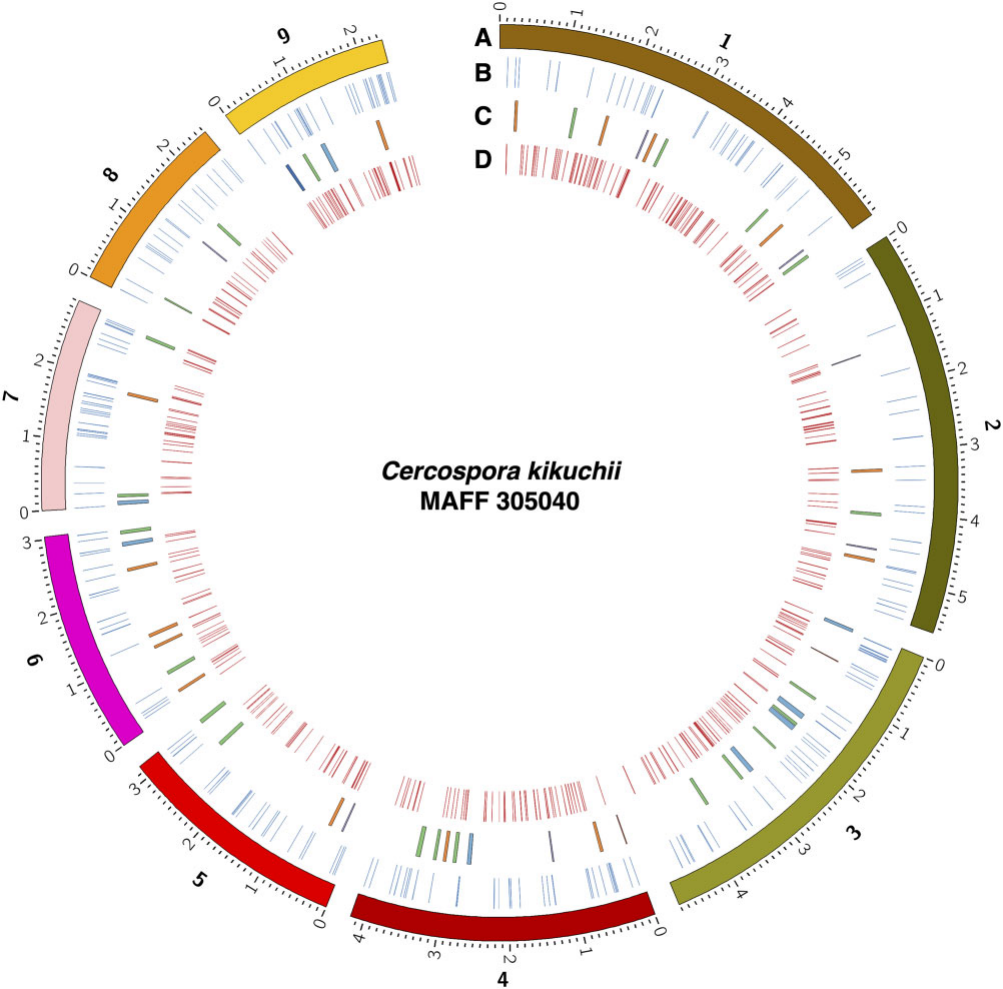
Circos plot of MAFF 305040 genome. Tracks indicates: (A) Contigs of MAFF 305040, minor ticks indicate 0.1 Mb. Numbers for tracks indicate identifier for contigs (1, contig_00001; 2, contig_00002; 3, contig_00003; 4, contig_00004; 5, contig_00005; 6, contig_00006; 7, contig_00007; 8, contig_00008; and 9, contig_00009); (B) Positions of the genes encoding effector candidates (blue); (C) Positions of the SM gene clusters. Colors indicate predicted SM type of the cluster: orange, polyketide; green, nonribosomal peptide; red, siderophore; purple, terpene and beta-lactone; and dark blue, ribosomally synthesized and post-translationally modified peptide. Other clusters are colored blue. (D) Positions of the genes encoding CAZymes (red).

In some plant pathogenic fungi, specific genomic compartments can function as toolboxes for effector genes. For example, in *Fusarium oxysporum*, effector genes such as *SIX* (*Secreted In Xylem*) are enriched in small dispensable chromosomes (approximately 2 Mb). Transmission of the chromosomes between the isolates enables the acquisition of pathogenicity to nonpathogens (Ma *et al.* 2010). For the genome of *C*. *kikuchii* MAFF 305040, the effector candidate genes were distributed among the nine contigs (Figure 1B). However, the frequency of the genes for effector candidates was different among the contigs. For the largest contig_00001, the frequency of the genes for effector candidates in 100 kb of nucleotide sequences was 0.60 (35 genes in 5,855,908 bp). Meanwhile, the frequency was more than double (1.23, 29 genes in 2,357,730 bp) for contig_00009. It is assumed that the benefit of effector-enriched chromosomes is a facile adaptation to environmental changes (Croll and McDonald 2012). Namely, conditionally dispensable small chromosomes fulfill the role of the toolbox for pathogenicity-related factors and accelerate changes in the pathogenicity by altering the content and/or expression profile of the effector genes on the chromosomes (Seidl *et al.* 2016). It is uncertain whether such genomic regions or chromosomes are in the *C*. *kikuchii* genome. Further studies of the gene composition of each chromosome and the virulence functions of the predicted effector candidates are necessary to define the properties of chromosomes.

SM gene clusters were also predicted in the nine contigs (Figure 1C, Table 2). A total of 55 gene clusters were predicted from the genome (Table 2). Numbers of each metabolite types from the cluster are also listed in Table 2. For instance, 15 polyketides, 22 nonribosomal peptides, and 6 terpenes were predicted. A cluster harboring multiple core biosynthetic genes was grouped into “others” in Table 2.

**Figure 2:**
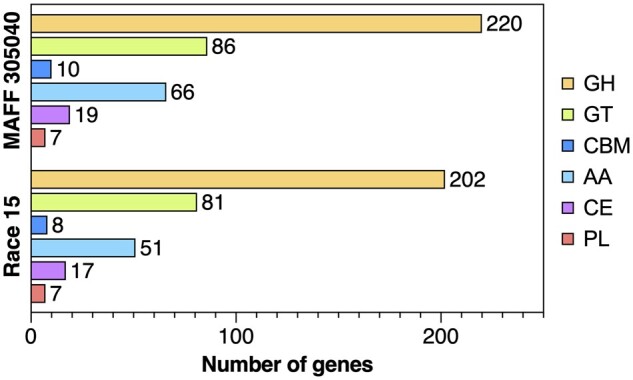
Number of genes in the *C*. *kikuchii* MAFF 305040 genome and *C*. *sojina* Race 15 genome for each CAZyme category. GH, glycoside hydrolase; GT, glycosyltransferase; CBM, carbohydrate-binding module; AA, auxiliary activity; CE, carbohydrate esterase; PL, polysaccharide lyase.

Carbohydrate-active enzymes (CAZymes) are also important for fungi, for successful infections of their plant hosts. CAZymes are used to degrade plant polysaccharides to obtain nutrients and to enhance the infection in the host (Zhao *et al.* 2014). A total of 399 genes encoding CAZymes were predicted by dbCAN and classified into six categories (Figures 1D and 2, Table 2). Some of the proteins were matched multiple categories. Among these, 220 genes were categorized as glycoside hydrolase (GH), 86 as glycosyltransferase (GT), 10 as carbohydrate-binding module (CBM), 66 as auxiliary activity (AA), 19 as carbohydrate esterase (CE), and 7 as polysaccharide lyase (PL).

The genomic composition of CAZymes is key to understanding the infection strategy of plant pathogenic fungi (Zhao *et al.* 2014; Hane *et al.* 2020). As described, different species of the genus *Cercospora* can utilize soybeans as their hosts. To understand their difference, their CAZyme profiles were compared. The CAZymes in the *C*. *sojina* Race 15 genome were also checked for this purpose. A total of 13,253 genes were predicted from 12 chromosomes of Race 15 by BRAKER2-trained Augustus. Then, the dbCAN meta server selected 359 genes as having CAZyme encoding sequences. This number is higher than in previous *C*. *sojina* genome analyses (number of CAZyme genes was 340, Gu *et al.* 2020). Interestingly, the number of genes encoding CAZymes in the *C*. *kikuchii* genome was greater than in *C*. *sojina* (Figure 2). As described, *C*. *kikuchii* has a smaller genome (34.44 Mb) than *C*. *sojina* (40.12 Mb, Gu *et al.* 2020). For instance, the number of genes coding for the GH family enzyme was different between the two genomes. The genome of *C*. *kikuchii* has some additional genes cording GH (Figure 2 and Table 3). It is known that some enzymes belonging to the GH family are critical for the infection of the host plant. For example, PsGH7a (GH7) contributes for virulence in soybean oomycete pathogen *Phytophthora sojae* (Tan *et al.* 2020). Based on the previous report (Zhao *et al.* 2014), 61 genes from *C*. *kikuchii* MAFF 305040 were grouped into plant cell wall (PCW in Table 3) as substrate of the encoding enzyme. For *C*. *sojina* Race 15, 53 genes were grouped into PCW (Table 3). Functional analysis of their pathogenicity-related factors, such as CAZymes, is needed for further study.

**Table 3:** Number of genes related to GH families identified from *C*. *kikuchii* MAFF 305040 and *C*. *sojina* Race 15

**Family** *^a^*	MAFF 305040	Race 15	**Substrate** *^b^*
GH64	4	4	CW (β-1,3-glucan)
GH1	3	3	CW (β-glycans)
GH2	6	6	CW (β-glycans)
GH3*	17	16	CW (β-glycans)
GH5*	14	13	CW (β-glycans)
GH32	3	3	ESR (sucrose/inulin)
GH37	2	2	ESR (trehalose)
GH65	1	1	ESR (trehalose)
GH15*	2	1	ESR (α-glucans)
GH30	2	2	FCW
GH85	1	1	FCW
GH18*	6	5	FCW (chitin)
GH20	1	1	FCW (chitin)
GH76	9	9	FCW (chitin)
GH17	4	5	FCW (β-1,3-glucan)
GH55	5	5	FCW (β-1,3-glucan)
GH71	2	2	FCW (β-1,3-glucan)
GH72	6	6	FCW (β-1,3-glucan)
GH81	1	1	FCW (β-1,3-glucan)
GH16*	11	9	FCW (β-glycans)
GH13	15	15	FCW + ESR (α-glucans)
GH7	1	1	PCW (cellulose)
GH12	1	1	PCW (cellulose)
GH10*	4	3	PCW (hemicellulose)
GH11	3	3	PCW (hemicellulose)
GH27	2	3	PCW (hemicellulose)
GH29*	3	2	PCW (hemicellulose)
GH35*	2	1	PCW (hemicellulose)
GH36*	2	1	PCW (hemicellulose)
GH39	0	1	PCW (hemicellulose)
GH51*	3	2	PCW (hemicellulose)
GH53	1	1	PCW (hemicellulose)
GH54	1	1	PCW (hemicellulose)
GH62	1	1	PCW (hemicellulose)
GH67	1	1	PCW (hemicellulose)
GH93*	1	0	PCW (hemicellulose)
GH115	1	2	PCW (hemicellulose)
GH43*	12	10	PCW (pectin + hemicellulose)
GH28	5	5	PCW (pectin)
GH78*	3	2	PCW (pectin)
GH88*	2	1	PCW (pectin)
GH105	3	3	PCW (pectin)
GH38	1	1	PG (N-/O-glycans)
GH47	9	9	PG (N-/O-glycans)
GH63	1	1	PG (N-glycans)
GH125	3	3	PG (N-glycans)
GH31*	9	8	PG + ESR + PCW (hemicellulose)
GH33	1	1	NA
GH42	1	1	NA
GH79	3	4	NA
GH92	7	7	NA
GH95*	2	0	NA
GH97*	1	0	NA
GH106	1	1	NA
GH114	1	1	NA
GH127	1	1	NA
GH128*	2	1	NA
GH130	1	1	NA
GH131	1	1	NA
GH132	1	1	NA
GH135	1	2	NA
GH139*	1	0	NA
GH141*	1	0	NA
GH142	1	1	NA
GH152	1	1	NA
GH154*	2	1	NA

a Asterisk indicates that the number of genes identified from the *C*. *kikuchii* MAFF 305040 was greater than the number identified from *C*. *sojina* Race 15.

b Information on the substrate was obtained from Zhao *et al.* (2014). CW, cell wall; ESR, energy storage and recovery; FCW, fungal cell wall; PCW, plant cell wall; PG, protein glycosylation; NA, not assigned.

As described, several *Cercospora* species, including cryptic groups, are known to cause diseases in soybeans. The ability to elucidate the relationships between these pathogens provides a strong incentive to define their diversity and classification. This is the first chromosome-level assembly for *C*. *kikuchii*, causal agent of CLB and PSS. Our highly contiguous genome assembly of *C*. *kikuchii* and the results obtained in this study provide a solid foundation for genome analysis of the genus *Cercospora*.
