# Erratum to: High-quality genome assembly of the soybean fungal pathogen *Cercospora kikuchii*

**DOI:** 10.1093/g3journal/jkab323

**Published:** 2021-11-28

**Authors:** Takeshi Kashiwa, Tomohiro Suzuki

*G3: Genes|Genomes|Genetics*, 2021, https://doi.org/10.1093/g3journal/jkab277

Due to a production error, several errors were inadvertently introduced into the above mentioned article. The necessary corrections have since been made. The publisher apologizes for the error.

